# Depressive symptoms as independent correlates of epilepsy‐related cognitive burden

**DOI:** 10.1002/epi.70108

**Published:** 2026-01-28

**Authors:** Biagio Maria Sancetta, Giuli Lippa, Marianna Nesta, Lorenzo Ricci, Simona Paola Carbone, Lorenzo Veronese, Giulia Conti, Marco Sferruzzi, Vincenzo Di Lazzaro, Mario Tombini, Giovanni Assenza

**Affiliations:** ^1^ Research Unit of Neurology, Department of Medicine and Surgery Università Campus Bio‐Medico di Roma Rome Italy; ^2^ Operative Research Unit of Neurology Fondazione Policlinico Universitario Campus Bio‐Medico Rome Italy

**Keywords:** cognitive burden, depressive symptoms, epilepsy, EpiTrack

## Abstract

**Objective:**

This study was undertaken to assess the relationship between the severity of depression and anxiety symptoms and epilepsy‐related variables and cognitive burden in people with epilepsy (PwE), as assessed using EpiTrack.

**Methods:**

We prospectively enrolled a cohort of PwE who underwent EpiTrack and evaluation by Generalized Anxiety Disorder‐7 (GAD‐7) and Beck Depression Inventory‐II (BDI‐II) scales. We assessed the correlation strength between EpiTrack, GAD‐7, BDI‐II, and the other clinical variables. Analysis of variance and covariance assessed the existence of GAD‐7/BDI‐II differences between PwE with and without epilepsy‐related cognitive impairment. Hierarchical regression analysis (HRA) and logistic regression were performed to characterize the association between BDI‐II and GAD‐7 and the presence/severity of epilepsy‐related cognitive burden. Mediation analysis was performed to evaluate the relationship between EpiTrack and the other variables applied.

**Results:**

We enrolled 100 PwE (42 ± 18 years old). EpiTrack inversely correlated with BDI‐II (−.33, *p* = .003, not with GAD‐7), seizures (−.34, *p* < .001), epileptiform abnormalities (34, *p* < .001), and pharmacological burden (.21, *p* = .002). BDI‐II correlated with seizure frequency (.27, *p* = .02). PwE with cognitive impairment had significantly higher BDI‐II scores, independently of age, seizure frequency, epileptiform abnormalities, and pharmacological load (*p* = .02–.04). BDI‐II effect on EpiTrack was independent from seizures. The addition of BDI‐II in HRA and logistic regression provided a significant increase of the *R* ^2^ value (*p* = .004) and of area under the curve (*p* = .02).

**Significance:**

More severe depressive symptoms are strongly associated with worse cognitive performance in PwE, independently of the other epilepsy‐related variables. Depressive symptoms could either forecast the occurrence of epilepsy‐related cognitive impairment or arise as a consequence of cognitive dysfunction in PwE. We confirmed the association between epilepsy severity and epilepsy‐related cognitive impairment.

**Key points**

- More severe depressive symptoms are associated with worse cognitive performance in epilepsy.
- People with epilepsy with cognitive impairment have greater depressive symptoms, independently of other clinical variables.
- Pharmacological burden, seizures, and epileptiform abnormalities are associated with worse cognitive performance.

## 1 INTRODUCTION

Epilepsy is one of the most common chronic neurological disorders, affecting approximately 50 million people worldwide.^1^, ^2^ At least one third of people with epilepsy (PwE) experience cognitive impairment,^3^ which can lead to significant social consequences, including academic difficulties, lower educational attainment, and unemployment.^4^ These aspects highlight the importance of understanding the factors that influence cognitive profiles for effective clinical management of PwE. The determinants of cognitive impairment in PwE are multifactorial.^3^ One of the most relevant factors affecting cognitive performance in PwE is the pharmacological burden.^5^ The current literature clearly indicates that antiseizure medications (ASMs) primarily impact attention and executive functions in PwE.^6^

The EpiTrack test is a validated tool for screening and investigating cognitive impairment in epilepsy.^7^ The clinical utility of EpiTrack in everyday clinical practice is well established.^8^, ^9^ Previous works largely explored risk factors for the development of cognitive impairment in PwE.^7^, ^8^

However, only a few studies have specifically explored the factors affecting the EpiTrack score,^9^, ^10^, ^11^ and even fewer have examined the relationship between the EpiTrack score and depressive and anxiety symptom severity.^12^ Some aspects of the determinants of epilepsy‐related cognitive burden are still unexplored and not completely understood. In particular, to the best of our knowledge, none of the studies conducted to date has explored the mutual and interconnected relationships between seizure‐related variables, the severity of anxiety and depressive symptoms, and cognitive performance.

Therefore, we designed the following study aiming to fill this knowledge gap. Specifically, we conducted a comprehensive, multimodal assessment of clinical and psychological variables to gain deeper insight into the factors associated with epilepsy‐related cognitive burden, with particular focus on anxiety and depression symptomatology. To reach this goal, (1) we collected clinical information and psychological scales for rating depressive and anxiety symptoms of a cohort of PwE who underwent prompt EpiTrack testing; (2) we tested the correlation strength and the mutual relationship between the variables collected and the EpiTrack score through moderation analysis; and (3) we performed regression analysis to test and compare the ability of psychological and epilepsy‐related factors in explaining EpiTrack score variance.

Better insight into this topic could have remarkable effects on clinical practice, because it may lay the foundation for more tailored therapeutic strategies in epilepsy, aimed at minimizing epilepsy‐related cognitive burden and the psychological consequences of cognitive impairment in PwE.

## 2 MATERIALS AND METHODS

This study was approved by the ethics committee (CET Lazio Area 2). Written informed consent was obtained from all participants. All procedures were under the ethical standards of the 1964 Declaration of Helsinki and its later amendments.

### 2.1 Enrollment

We performed a monocentric, cross‐sectional, prospective, observational study in the outpatient epilepsy centers at Fondazione Policlinico Campus Bio‐Medico from September 2022 to January 2025.

Inclusion criteria were as follows: (1) epilepsy diagnosis according to the current International League Against Epilepsy (ILAE) working definition,^13^ (2) age = 18–65 years, and (3) clinical standard electroencephalographic (EEG)/video‐EEG acquisition in the morning before clinical scales administration (see later in the text).

Exclusion criteria were as follows: (1) severe cognitive/motor disabilities not allowing EpiTrack testing completion, (2) clinical seizure(s) in the 24 h before clinical scale administration, (3) assumption of neuroactive medications other than ASMs, (4) history of progressive/degenerative neurological diseases and/or severe psychiatric disorders, and (5) excessive sleepiness before clinical scale administration.

### 2.2 Data acquisition

#### 2.2.1 Clinical scales acquisition

The following clinical scales were administered by a trained operator in the morning (same timeslot, between 9 and 11 a.m.) in a distraction‐free outpatient setting:

1. Generalized Anxiety Disorder‐7 (GAD‐7):^14^ a seven‐item scale rated on a 4‐point Likert‐type scale and used to screen generalized anxiety disorder severity. Score ranges from 0 to 21. Anxiety level can score as follows: absent anxiety (0–4), mild anxiety (5–9), moderate anxiety (10–14), and severe anxiety (15–21).
2. Beck Depression Inventory II (BDI‐II):^15^ a 21‐item scale used to evaluate the presence of depressive symptoms. Score ranges from 0 to 63. Level of depression can be scored as follows: absent depression (0–13), mild depression (14–19), moderate depression (20–28), and severe depression (29–63).
3. EpiTrack test^16^, ^17^: used to screen cognitive impairment in PwE. It is composed of six items exploring the most impaired cognitive domain in PwE due to ASMs, that is, attention, memory, executive functions, speed of thought, verbal fluency, and visual–spatial skills.^5^ Score ranges from 4 to 42. Global EpiTrack score can be corrected by age. Based on the age‐corrected EpiTrack score, the following performance categories can be identified: cognitive impairment (≤31 points) and noncognitive impairment (≥32 points).

All PwE with depressive symptomatology were promptly treated with a personalized strategy (pharmacological intervention, psychiatric evaluation, and/or cognitive/behavior therapy) after clinical scale acquisition.

#### 2.2.2 Clinical data acquisition

We collected the following variables for each PwE: (1) demographic features (age and sex); (2) features related to epilepsy history, that is, age at epilepsy onset and duration, prevalence of sporadic interictal epileptiform abnormalities (IEAs)^18^ at EEG recording, seizure frequency (mean number of seizures in the past 3 months), presence of drug‐resistant epilepsy (DRE) according to the current ILAE definition,^19^ and epilepsy type (focal unknown, focal structural, generalized or unknown onset)^20^ ^;^ and (3) information related to epilepsy treatment, that is, number and types of ASMs (Na‐blocker, synaptic vesicle glycoprotein 2A target, γ‐aminobutyric acidergic, and glutamate antagonist)^21^ and defined daily dose (DDD).^22^ Based on seizure frequency, we categorized our cohort into rare (≤1 seizure/month), occasional (1–3 seizures/month), or frequent seizures (>3 seizures/month).^20^

### 2.3 Statistical analysis

Statistical analysis was conducted using Python 3.11. The normality of data was checked using Shapiro–Wilk test. Nonnormally distributed data were reported by median and interquartile ranges, whereas normally distributed data were reported by mean ± SD. Results of hypothesis tests were expressed through *p*‐value and size effects (via Cohen *d*). Categorical variables were compared using the χ^2^ test, whereas parametric (Student *t*‐test) and nonparametric (Mann–Whitney test) tests as appropriate were used for continuous variables.

Correlation analysis was performed to check the strength and significance of the correlation between EpiTrack score and the other variables and scales collected. Spearman correlation was performed for nonnormally distributed variables, whereas Pearson correlation was performed for normally distributed variables. Results of correlations were expressed by the correlation coefficient *r*, the associated *p*‐value, and the 95% confidence intervals (CIs).

To compare BDI‐II or GAD‐7 according to EpiTrack performance categories, we performed analysis of covariance (ANCOVA) with EpiTrack categories (two levels: impairment and nonimpairment) as between‐subjects factor and clinical variables with a significant correlation with EpiTrack score as covariates and analysis of variance (ANOVA) with EpiTrack categories as first between‐subjects factor and number of ASMs (three levels: 1 ASM, 2 ASM, and ≥3 ASM), seizure frequency (three levels: rare, occasional and frequent), or IEA frequency (three levels: <1/min, 1/min–1/10 s, >1/10 s) as second between‐subjects factor. The normality distribution assumption for ANOVA was checked through residual Q‐Q plots. Log or square root transformations (as appropriate) were applied when necessary. The homoscedasticity assumption was checked through Barlett test.

To better explore the mutual relationship between EpiTrack score and the other variables collected, we performed a mediation analysis through the four‐step algorithm of Baron and Kenny,^23^ to ensure comparability with previous studies in the field of epilepsy, and provide a transparent, step‐by‐step illustration of the mediation process.^24^, ^25^, ^26^ Results were expressed through the *p*‐values of average causal mediation effects (ACMEs), average direct effects (ADEs), and total effects (TEs). We performed bootstrapping (iterations = 5000) for significance testing.^27^

Subgroup analysis was performed separately in the DRE and non‐DRE groups to take into account possible influences of DRE conditions on our main findings. Alpha level was set at .05. Benjamini–Hochberg's false discovery rate procedure was used to correct for multiple comparisons, both in the post hoc tests and across the broader set of analyses, to reduce type I error. All *p*‐values were reported after corrections.

### 2.4 Regression analysis

#### 2.4.1 Linear regression

Linear regression (ordinary least squares method) was used to describe the variance of global EpiTrack score through the variables collected. The contribution of anxiety and depressive symptoms (BDI‐II and/or GAD‐7) to the variance in EpiTrack scores was examined using a simple linear regression model. Multiple linear regression model construction was initialized by fitting the model with (1) qualitative variables with a significant difference between PwE with and without cognitive impairment and (2) quantitative variables with a significant correlation with EpiTrack score.

A backpropagation elimination algorithm was used to identify the optimal exploratory variables for explaining EpiTrack score variance and to prevent multicollinearity issues. During tuning, all variables with *p* > .05 were gradually removed starting from those with the highest *p*‐value. Coefficient tuning stopped when all linear coefficients (βs) significantly differed from zero. Additionally, the variance inflation factor was calculated for the selected exploratory variables.

Hierarchical regression model was used to assess the significance of psychological variables in explaining EpiTrack score variance when compared to the other exploratory variables. Variables were entered into the model in consecutive steps; BDI‐II and/or GAD‐7 were added in the final stage as main variable of interest. Comparison of models was performed with ANOVA.

#### 2.4.2 Logistic regression

Logistic regression analysis was conducted to evaluate the ability of exploratory variables to distinguish PwE based on EpiTrack performance categories. Model was constructed with the variables that survived to backpropagation elimination algorithm (see the previous section).

Logistic regression was performed twice: once including all exploratory variables except BDI‐II and GAD‐7 (REG_BDI‐II/GAD‐7‐_) and once including BDI‐II and GAD‐7 (REG_BDI‐II/GAD‐7+_). Results of logistic regression were validated through fivefold cross‐validation to prevent overfitting. Receiver operating characteristic (ROC) curves and their associated area under the curve (AUC) were calculated. Sensitivity, specificity, positive predictive value (PPV), negative predictive value (NPV), and accuracy of REG_BDI‐II/GAD‐7+_ were obtained. For each metric, we reported the median value ± SD obtained after cross‐validation procedure.

Permutation testing (number of iterations = 1000) was performed on REG_BDI‐II/GAD‐7‐_ and REG_BDI‐II/GAD‐7+_ to assess the significance of BDI‐II addition for discriminating PwE with cognitive impairment as assessed by EpiTrack. Results of permutation were expressed by reporting the most frequent *p*‐value obtained after meta‐permutation analysis.^28^, ^29^, ^30^

## 3 RESULTS

### 3.1 Clinical characteristics

Clinical characteristics of our cohorts are displayed in Table 1. In total, we enrolled 100 PwE (42.26 ± 18.16 years old, 50 females); among these, 55 PwE had cognitive impairment as assessed by EpiTrack test. Regarding clinical scales, PwE with cognitive impairment had significantly higher BDI‐II scores (*p* < .001, *d* = .69). Concerning demographic features and features related to epilepsy history, PwE with cognitive impairment had significantly higher female‐to‐male ratio (*p* = .04), higher rate of PwE with frequent IEAs (*p* < .001), lower rate of PwE with absent and occasional IEAs (*p* = .04), and higher seizure frequency (*p* = .002, *d* = .48). Concerning information related to epilepsy treatment, PwE with cognitive impairment had higher DDD (*p* = .01, *d* = .27) and higher number of Na‐blocking ASMs (*p* < .001, *d* = .62). BDI‐II levels and EpiTrack score did not statistically differ according to ASM types. Female‐to‐male ratio was significantly higher in PwE with depressive symptoms (BDI‐II score > 13 points) compared to those without depression (24/15 vs. 27/34, *p* = .008).

**TABLE 1:** Clinical characteristics of our cohort in aggregated form and results of hypothesis tests between PwE with cognitive impairment and those without cognitive impairment, as assessed by the EpiTrack test.

Characteristic	Impaired	Not impaired	*p* (Cohen *d*)
PwE, *n*	55	45	
Clinical scales			
Global EpiTrack score, points, median (IQR) [CI]	18.5 (9) [17.8–21]	31 (5) [30.8–32.7]	<.001 (1.48)
Age‐corrected EpitTrack score, points, median (IQR) [CI]	21 (8) [20.2–23]	34 (4.5) [32.1–36.5]	<.001 (1.46)
BDI‐II score, points, median (IQR) [CI]	11 (10.5) [4.6–9.2]	5 (8.5) [9.3–15.6]	<.001 (.62)
GAD‐7 score, points, median (IQR) [CI]	6 (7.75) [2.8–7.2]	5 (5) [3.8–9.2]	n.s.
Demographic features			
Sex, F/M ratio	31/24	19/26	.05
Age, years, mean ± SD [CI]	41.14 ± 19.83 [39.2–50.5]	39.96 ± 16.53 [35.1–44.3]	n.s.
Features related to epilepsy history			
Epilepsy onset, years, mean ± SD [CI]	39.86 ± 22.54 [35.6–46.2]	32.24 ± 25.72 [25.1–39.4]	n.s.
Epilepsy duration, years, mean ± SD [CI]	15.21 ± 16.33 [10.6–19.8]	13.18 ± 14.62 [9.1–17.2]	n.s.
IEA frequency, *n* (%)			
Absent	12 (26.67%)	22 (40%)	.04
Rare	4 (8.89%)	9 (16.36%)	n.s.
Occasional	11 (24.44%)	21 (38.18%)	.04
Frequent	14 (31.11%)	3 (5.45%)	<.001
Abundant	2 (4.44%)	0	n.s.
Seizure frequency, *n*/3 months, median (IQR) [CI]	1.5 (4.25) [.7–2.5]	0 (1.25) [1.6–10.1]	.002 (.48)
Epilepsy type, *n* (%)			
Focal structural	7 (12.73%)	7 (15.56%)	n.s.
Focal unknown origin	34 (61.82%)	33 (73.33%)	n.s.
Generalized onset	4 (7.27%)	5 (11.11%)	n.s.
DRE, *n* (%)	19 (34.55%)	13 (28.11%)	n.s.
Information related to epilepsy treatment			
ASMs, *n*, median (IQR)	1 (1)	2 (1.25)	n.s.
DDD, mean ± SD [CI]	6.04 ± 1.51 [5.6–7.5]	1.38 ± 1.09 [1.1–1.7]	.01 (.27)
Type of ASM, *n* (%)			
Na blocker	37 (62.67%)	39 (86.67%)	<.001
GABA agonist	22 (40%)	19 (42.22%)	n.s.
GLUT antagonist	7 (12.73%)	9 (20%)	n.s.
SV2A	19 (34.55%)	20 (44.44%)	n.s.

Abbreviations: ASM, antiseizure medication; BDI‐II, Beck Depression Inventory II; CI, confidence interval; DDD, defined daily dose; DRE, drug‐resistant epilepsy; F/M, female‐to‐male; GABA, γ‐aminobutyric acid; GAD‐7, Generalized Anxiety Disorder‐7; GLUT, glutamate; IEA, interictal epileptiform abnormalityies; IQR, interquartile range; n.s., not significant; PwE, people with epilepsy; SV2A, synaptic vesicle glycoprotein 2A.

### 3.2 Correlation analysis

**FIGURE 1:**
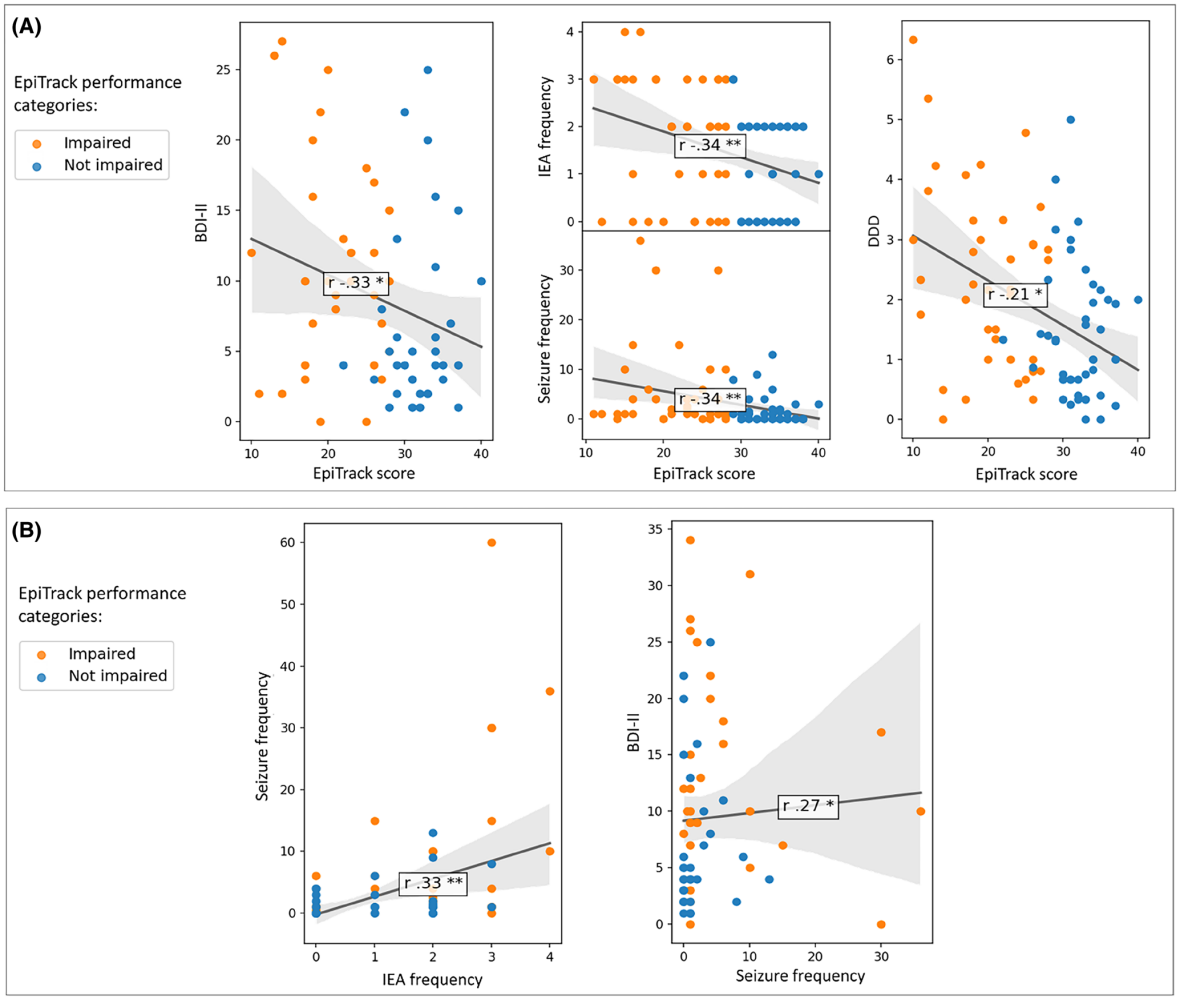
Results of correlation analysis between age‐corrected EpiTrack score and the other variables (A) and between seizure frequency, Beck Depression Inventory II (BDI‐II) score, and interictal epileptiform abnormality (IEA) frequency (B). Orange dots represent people with epilepsy with cognitive impairment as assessed by Epitrack, whereas blue dots represent those without cognitive impairment. For the sake of clarity, we only showed the correlation result concerning the age‐corrected EpiTrack score. DDD, defined daily dose; IEAs, interictal epileptiform abnormalities; *r*, correlation coefficient. *p < .05, **p < .001.

Age‐corrected EpiTrack score was inversely correlated with BDI‐II (*r* = −.33, *p* = .003, 95% CI = −.56 to −.13), seizure frequency (*r* = −.34, *p* < .001, 95% CI = −.51 to −.18), IEA frequency (*r* = −.34, *p* < .001, 95% CI = −.59 to −.12), DDD (*r* = −.21, *p* = .002, 95% CI = −.49 to −.11), and ASM number (*r* = −.38, *p* < .001, 95% CI = −.54 to −.17). BDI‐II was directly correlated with seizure frequency (*r* = .27, *p* = .02, 95% CI = .05 to .47) and the latter was directly correlated with IEA frequency (*r* = .33, *p* < .001, 95% CI = .12 to −51). BDI‐II was not statistically correlated with DDD or IEA frequency. GAD‐7 was not statistically correlated with EpiTrack score (Figure 1).

In the non‐DRE subgroup, the age‐corrected EpiTrack score was inversely correlated with BDI‐II, number of ASMs, seizure frequency, and IEA frequency; conversely, in the DRE subgroup, the age‐corrected EpiTrack score was inversely correlated with DDD, BDI‐II, and number of ASMs (for more details, see Supplementary Materials).

### 3.3 ANCOVA and ANOVA

**FIGURE 2:**
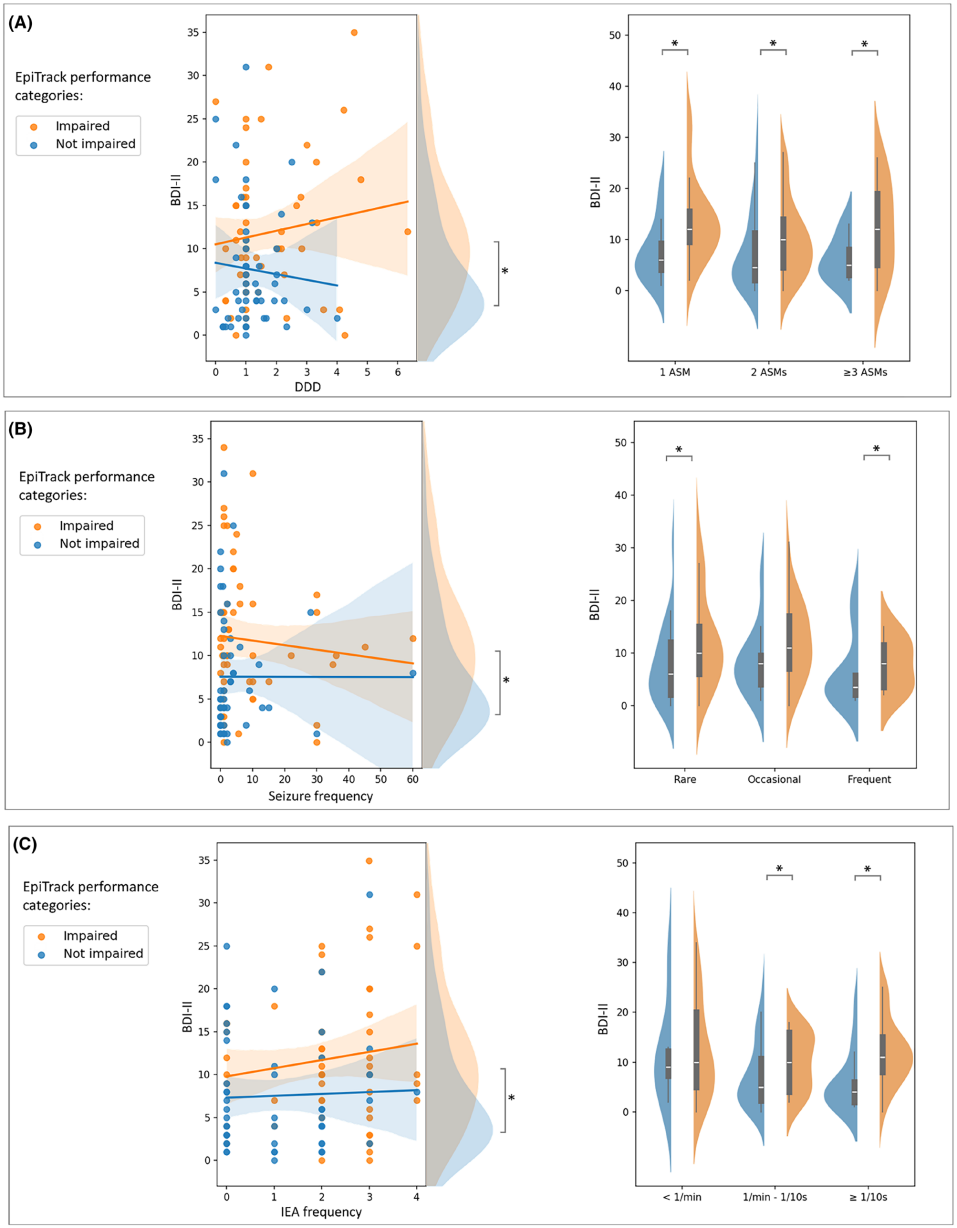
Results of analysis of covariance (left panels) and analysis of variance (right panels) on Beck Depression Inventory II (BDI‐II) levels with defined daily dose (DDD) and number of antiseizure medications (ASMs) as covariate and between‐subject factor, respectively (A), seizure frequency as covariate and between‐subject factor (B), and IEA frequency as covariate and between‐subject factor (C). Orange dots represent people with epilepsy with cognitive impairment as assessed by EpiTrack, whereas blue dots represent those without cognitive impairment. IEA, interictal epileptiform abnormality. *p < .05.

ANCOVA showed a significant between‐subject factor influence on BDI‐II variance both when covariates (DDD, IEAs, and seizure frequency) were added altogether in the model (*F* = 2.49, *p* = .03) and separately (for DDD: *F* = 7.48, *p* = .007; for IEA frequency: *F* = 5.31, *p* = .02; for seizure frequency: *F* = 8.82, *p* = .003). Similar results were obtained even in DRE and non‐DRE subgroups (Figure 2; for more details, see Supplementary Materials).

ANOVA showed a significant number of ASMs*EpiTrack categories interaction (*F* = 7.88, *p* = .006), IEA frequency*EpiTrack categories interaction (*F* = 5.56, *p* = .03), and seizure frequency*EpiTrack categories interaction (*F* = 3.99, *p* = .04). Post hoc analysis showed that, compared to PwE without cognitive impairment, PwE with cognitive impairment exhibited higher BDI‐II in all the categorial variables analyzed, with a significant difference in all ASM categories (*p* = .04, .02, .03; *d* = .39, 1.09, .43), in PwE with IEA frequency between 1/min and 1/10 s (*p* = .003, *d* = .63) and IEA frequency ≥ 1 IEA/10 s (*p* = .03, *d* = .47), and in PwE with rare (*p* = .02, *d* = 1.58) and frequent seizures (*p* = .04, *d* = .53).

### 3.4 Moderation analysis

**FIGURE 3:**
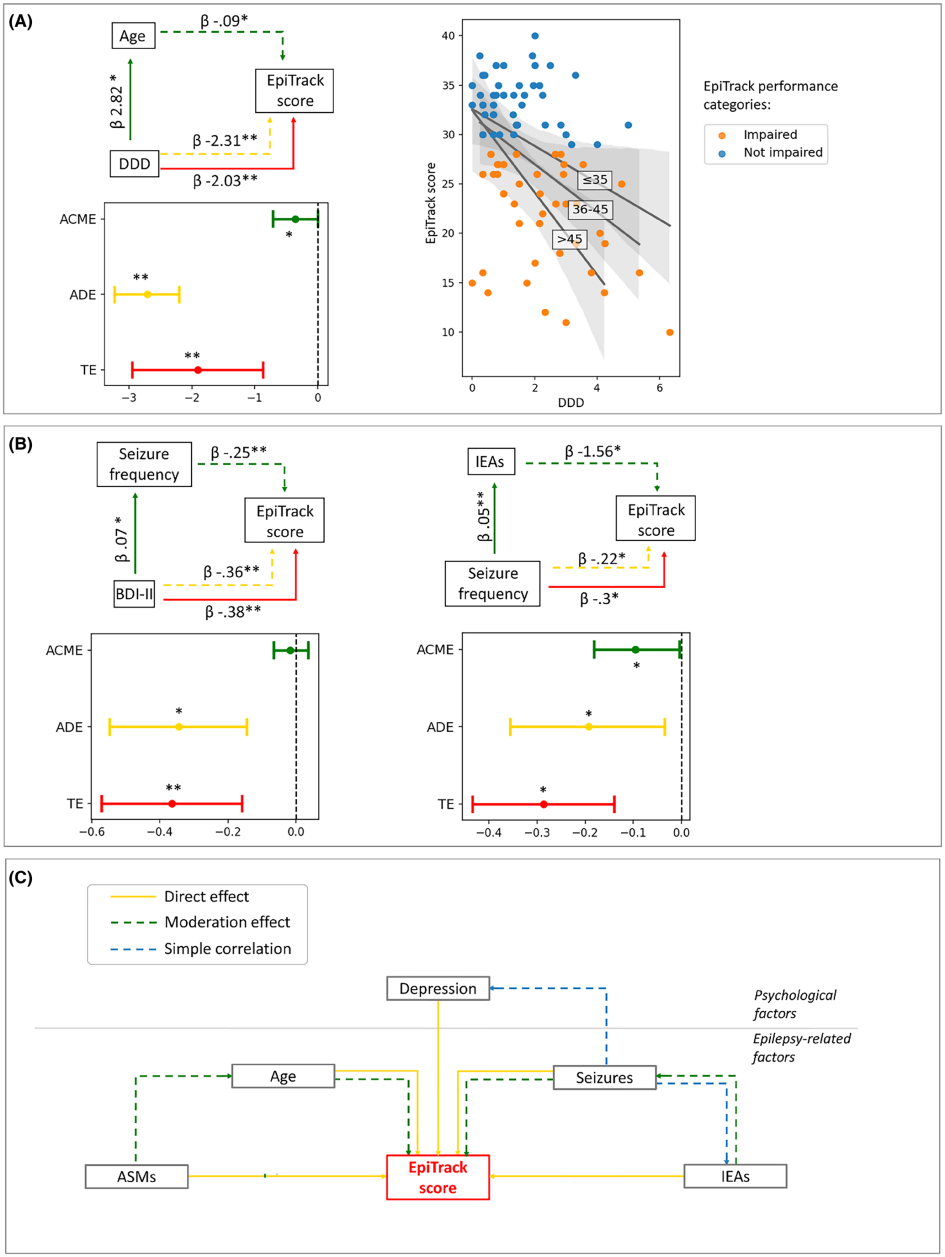
Results of mediation analysis. Red continuous arrows indicate the first step of moderation analysis (model: outcome ~ effector), green continuous arrows denote the second step of moderation analysis (model: mediator ~ effector), and dotted arrows (green arrows for moderator and yellow arrows for effector) represent the third step of moderation analysis (model: outcome ~ effector mediator). Interval plots show mediation analysis results after bootstrapping (total effect [TE] in red, average direct effect [ADE] in yellow, and average causal mediation effect [ACME] in green). (A) Defined daily dose (DDD) as effector, age as moderator, and age‐corrected EpiTrack score as effector (left plot). Scatterplot (age‐corrected EpiTrack score ~ DDD) reflects slopes of people with epilepsy (PwE) ≤35 years, PwE 36–45 years, and PwE 46–65 years old (right plot). (B) Beck Depression Inventory II (BDI‐II) as effector, seizure frequency as moderator, and age‐corrected EpiTrack score as effector (left plot). Seizure frequency as effector, interictal epileptiform abnormality (IEA) frequency as moderator, and age‐corrected EpiTrack score as effector (right plot). (C) Diagram displaying the main factors affecting the EpiTrack score and their interconnected relationship based on our main findings of correlation and moderation analysis. ASM, antiseizure medication; β, linear coefficient of the regression models. *p < .05, **p < .001.

DDD revealed a significant TE on EpiTrack score as well as a significant ADE when accounting for age (*p* < .001). Moderation analysis further revealed a significant ACME of age on the relationship between DDD and EpiTrack score (*p* = .05). Confirming this, for equal DDD value, PwE ≤35 years old exhibited a higher EpiTrack score compared to those 36–45 years and 46–65 years old (Figure 3).

BDI‐II showed a significant TE on EpiTrack (*p* < .001); BDI‐II effect on EpiTrack score remained significant when accounting for seizure frequency effect (ADE *p* = .004); BDI‐II effect on EpiTrack score was not moderated by an indirect influence of seizure frequency. Similar results were obtained even in DRE and non‐DRE subgroups (for more details, see Supplementary materials).

Finally, seizure frequency revealed a significant TE on EpiTrack score (*p* = .006), and its effect remained significant when accounting for IEA frequency (ADE *p* = .04); the analysis also evidenced a significant ACME of IEA frequency on the relationship between seizure frequency and EpiTrack score (*p* = .04).

### 3.5 Regression analysis

At the end of backpropagation elimination algorithm, the model included the following variables: BDI‐II, DDD, IEA frequency, and sex. The corresponding variance inflation factors were as follows: BDI‐II, 2.37; DDD, 2.27; IEA frequency, 2.55; and sex, 1.82. During the execution of hierarchical regression, DDD level was entered into the first stage of the models, followed by sex, IEA frequency, and BDI‐II. Table 2 summarizes the results of the simple linear regression model (EpiTrack ~ BDI‐II), hierarchical model, and final model (EpiTrack ~ DDD + IEA frequency + sex + BDI‐II). BDI‐II alone accounted for approximately 9% of EpiTrack score variance (*p* < .001). DDD, sex, and IEA frequency accounted for 27% of the variance in the outcome. BDI‐II level was found to increase significantly (adjusted *R* ^2^ = .37, *F* = 4.57, *p* = .004) the explanatory power of the final model when added to DDD, sex, and IEA frequency.

**TABLE 2:** Summary of hierarchical multiple regression.

Outcome variable: EpiTrack score	Model summary					Model comparison					
	*β*	SE	*t*	Pr| > *t*|	95% CI	*R* ^2^, Δ*R* ^2^	Adj. *R* ^2^, ΔAdj. *R* ^2^	ΔSSR	ΔDf	*F*	Pr|> *F*|
Simple model											
Exploratory variables											
BDI‐II	−.26	.09	−2.86	.005	−.46 to −.08	.09, –	.08, –	–	–	–	–
Hierarchical model											
Step 1											
Exploratory variables								–	–	–	–
DDD	−2.74	.52	−5.28	<.001	−3.77 to −1.71	.24, –	.02, –				
Step 2											
Exploratory variables											
DDD	−2.83	.5	−5.65	<.001	−3.83 to −1.84	.27	.25	402.79	1	8.17	.03
Sex	−1.85	1.3	−2.96	.04	−2.43 to −.27	.03	.05				
Step 3											
Exploratory variables											
DDD	−2.27	.52	−4.38	<.001	−3.3 to −1.24	.32, .05	.31, .06	375.11	1	8.75	.003
Sex	−1.58	1.26	−2.84	.03	−2.08 to −1.08						
IEA frequency	−2.08	.53	−2.96	.004	−2.56 to −1.39						
Step 4											
Exploratory variables											
DDD	−2.19	.51	−4.28	<.001	−3.21 to −1.18	.37, .05	.35, .04	173.58	1	4.57	.**004**
Sex	−1.42	1.24	−2.76	.007	−2.89 to −.97						
IEA frequency	‐ 1.57	.53	−2.68	.04	−2.48 to −.37						
BDI‐II	−1.82	.08	−2.04	.006	−2.33 to −.05						

Abbreviations: Adj., adjusted; BDI‐II, Beck Depression Inventory II; CI, confidence interval; DDD, defined daily dose; IEA, interictal epileptiform abnormality; Pr, probability; *β*, linear coefficient; ΔAdj‐*R* ^2^, models difference of adjusted *R* ^2^ values; ΔDf, difference of degrees of freedom; Δ*R* ^2^, models difference of *R* ^2^ values; ΔSSR, sum of squares difference.

**FIGURE 4:**
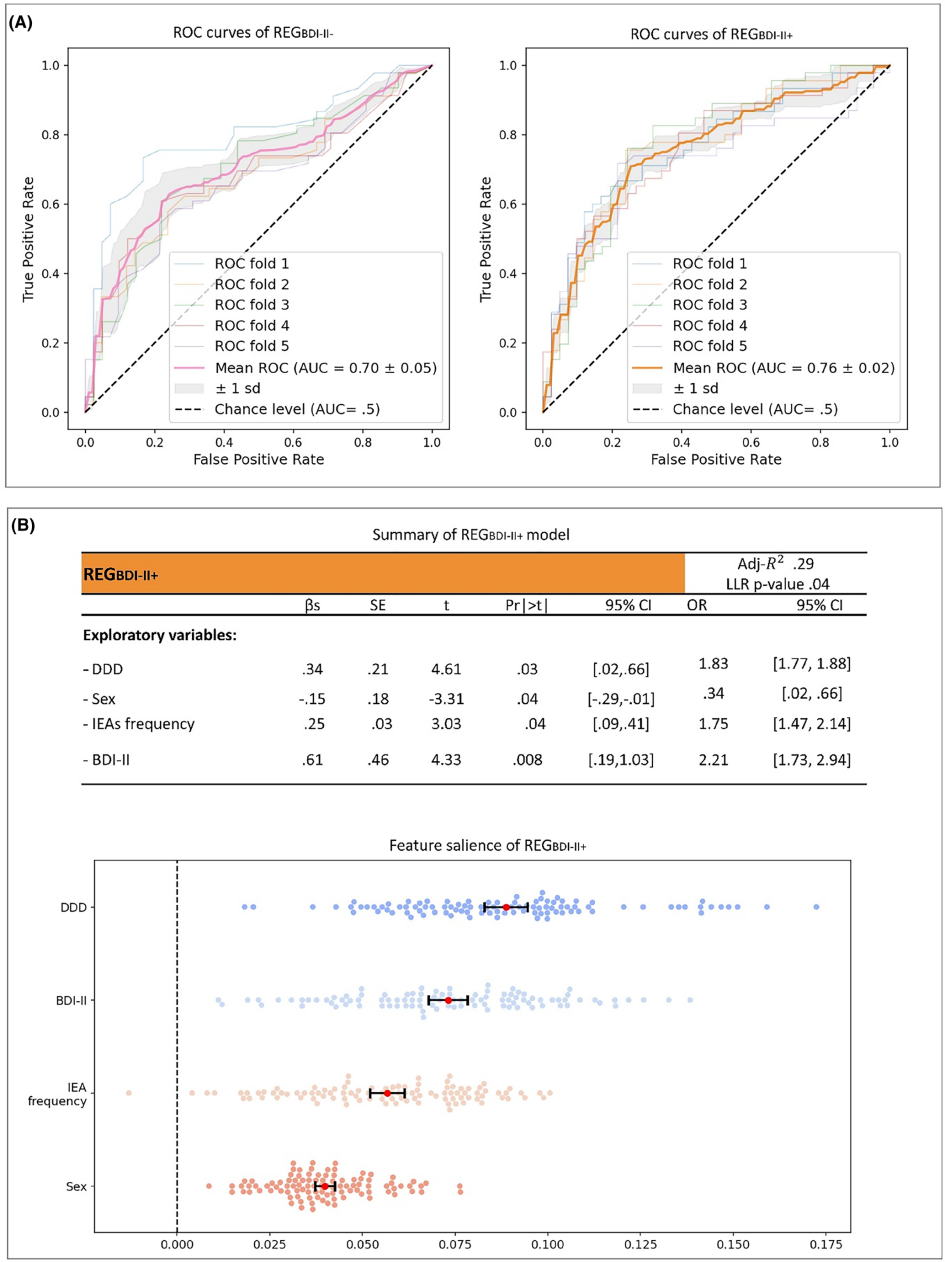
Main results of logistic regression analysis. (A) Receiver operating characteristic (ROC) curves and their associated areas under the curve (AUCs) of logistic regression models expressed by median AUC (pink curve for REG_BDI‐II‐_ and orange curve for REG_BDI‐II+_) ± 1 SD (in gray). In the legend, we reported the ROC curves' AUCs of every single fold of the cross‐validation procedure. (B) The upper panel displays the model summary of REG_BDI‐II_. The lower panel displays feature salience analysis of the REG_BDI‐II+_ model (without cross‐validation). Each dot represents the result of permutation feature importance after a single iteration (the difference between the ROC curve's AUCs before and after the shuffle for the current feature). Error bars display median value (red dots) ± 1 SD (black tails). Adj, adjusted; BDI‐II, Beck Depression Inventory II; CI, confidence interval; DDD, defined daily dose; IEA, interictal epileptiform abnormality; LLR, likelihood ratio test; OR, odds ratio; Pr, probability; REG_BDI‐II‐_, model trained without BDI‐II; REG_BDI‐II+_, model trained with BDI‐II; ROC, receiver operating characteristics; β, linear coefficient.

Results of logistic regression model on *RES*_*BDI‐II‐*_ (DDD + sex + IEA frequency) and *RES*_*BDI‐II+*_ (DDD + sex + IEA frequency + BDI‐II) are shown in Figure 4. The ROC AUC of *RES*_*BDI‐II+*_ (.76 ± .02) was significantly higher than that of *RES*_*BDI‐II‐*_ (difference in AUCs = .06, *p* = .02), indicating that including BDI‐II significantly improved the model's accuracy.

Performances of *RES*_*BDI‐II+*_ were as follows: sensitivity = .94 ± .06, specificity = .67 ± .08, PPV = .59 ± .01, NPV = .87 ± .03, and accuracy = .69 ± .02. The features with the highest influence on decision scores of *RES*_*BDI‐II+*_ were, in descending order, DDD (.09, 95% CI = .083–.095), BDI‐II (.07, 95% CI = .068–.078), IEA frequency (.06, 95% CI = .052–.061), and sex (.04, 95% CI = .037–.043).

## 4 DISCUSSION

In the present work, we performed a comprehensive evaluation of the factors associated with epilepsy‐related cognitive burden, as assessed by the EpiTrack test, with a particular focus on depressive symptoms. The main findings of this article can be summarized as follows: (1) depressive symptoms were significantly higher in PwE with cognitive impairment as assessed by EpiTrack, independently of the other ASM‐ and epilepsy‐related variables (DDD, seizures, and IEAs); (2) depressive symptoms strongly correlate with the occurrence of worse cognitive performance, without a significant moderation effect of the other epilepsy‐related variables; and (3) seizures, IEAs, and DDD directly influence the occurrence of epilepsy‐related cognitive impairment in PwE.

The most impaired cognitive domains in PwE are attention, memory, executive functions, speed of thought, verbal fluency, and visual–spatial skills.^31^ The impairment of these domains leads to significant social disabilities, such as inability to drive, great academic difficulties, a lower education level, and a higher unemployment rate.^4^, ^32^ Therefore, a better understanding of the factors impacting epilepsy‐related cognitive burden is essential for improving the balancing of ASM effectiveness and tolerability and, more generally, for improving the global well‐being of PwE. Our multimodal approach demonstrated a complex and mutual relationship between age, pharmacological burden, psychological factors, IEAs, seizures, and epilepsy‐related cognitive impairment in PwE, evidencing notable results never reported explicitly in current literature.

The major result that must be highlighted is the strong relationship between EpiTrack score and depressive symptoms. Similar to the previous work on this topic,^3^, ^12^, ^33^ we found different BDI‐II scores in PwE according to the EpiTrack performance categories, with PwE with cognitive impairment having significantly higher BDI‐II scores compared to those without cognitive impairment. First of all, the strong link between EpiTrack scores and depressive symptoms was reported by the inverse correlation between the two measures. Additionally, the association between depressive symptoms and cognitive dysfunction remained significant even after adjusting for epileptological clinical variables that were collinear with EpiTrack scores (IEAs, seizure frequency, and pharmacological burden), as demonstrated by ANOVA, ANCOVA, and mediation analyses. Moreover, including depressive symptom severity in the regression analysis provided significant added value for both explaining EpiTrack score variance and identifying PwE with cognitive dysfunction.

On the other hand, our findings confirmed that poorer cognitive performance is associated with greater epilepsy severity^34^; in turn, higher disease severity was associated with more severe depressive symptomatology.^35^, ^36^ However, the link between depressive symptoms and the occurrence of cognitive impairment seemed to be independent of epilepsy severity. The negative correlation between BDI‐II and EpiTrack remained significant when included together with seizure frequency, without a significant mediation effect of seizure frequency on the relationship between depressive symptomatology and EpiTrack score (as assessed through moderation analysis). Moreover, once again, ANCOVA and ANOVA showed distinct BDI‐II patterns according to EpiTrack performance categories, independently of seizure frequency. From this perspective, the higher female/male ratio in PwE with cognitive impairment could be justified by the higher BDI‐II scores in females of our cohort, widely reported in previous work about gender differences in depressive symptoms.^37^

Therefore, the association between EpiTrack and BDI‐II seemed to be independent of the other ASM‐ and epilepsy‐related variables involved. This strong relationship may be bidirectional. On one hand, depressive symptoms could independently contribute to the development of cognitive dysfunction in epilepsy, making PwE with more severe depressive symptomatology more susceptible to the cognitive consequences due to pharmacological treatments, IEAs, and seizures. On the other hand, PwE with greater cognitive impairment are more likely to develop depressive symptoms. Cognitively impaired PwE often experience reduced activity and poorer psychosocial functioning, which can increase depressive symptomatology. However, disentangling this complex relationship is challenging within the scope of the present study.

Regardless of the precise interpretation of our results, these findings carry important clinical implications. Our article strongly underscores the need for early recognition and management of depressive symptoms, which may either forecast the occurrence of epilepsy‐related cognitive impairment or arise as a consequence of cognitive dysfunction in PwE. Depressive symptoms are readily identifiable in clinical settings and can be effectively addressed through medical, cognitive, or behavioral interventions. Routine depression screening is therefore strongly recommended for clinicians managing PwE, as timely treatment can improve mood, daily functioning, and overall well‐being, while potentially minimizing the psychological impact of cognitive impairment.

Our work also bears out the strong association between EpiTrack, age, and DDD, with higher pharmacological burden and age associated with worse cognitive performance. The inverse association between age and cognitive performance is well documented, both in healthy subjects and in PwE.^38^, ^39^ Similarly, numerous studies have stressed the association between pharmacological burden and cognitive impairment in PwE.^3^, ^6^, ^40^ Interestingly, our analysis revealed that age directly mediated the relationship between DDD and cognitive performance. Specifically, the elderly seemed to be more susceptible to the pharmacological effect on cognitive performance. These results align with prior works showing increased vulnerability to the cognitive effects in the elderly of anticholinergic/sedative drug burden.^41^, ^42^, ^43^ Finally, our work confirms the substantial impact of seizures and IEAs on cognitive impairment, as evidenced by the inverse correlation between seizure and IEA frequency and EpiTrack scores, and by the higher frequency of both in PwE with cognitive impairment. Moreover, IEA effect on the development of cognitive dysfunction seemed both direct and indirect through a mediation effect on the relationship between seizure and EpiTrack score. These findings are not surprising. IEAs predispose to a higher probability of seizure occurrence,^44^ and the latter cause persistent changes in neuronal circuits manifesting as both psychological and cognitive disorders.^45^, ^46^ On the other hand, IEAs could directly affect cognitive performance in PwE due to persistent and chronic interference in cortical networks subtending physiological cognitive processes.^47^

All these findings confirm the substantial impact of epilepsy severity on the development of cognitive dysfunction in PwE. However, subgroup analyses did not replicate the correlations between EpiTrack scores and either seizure or IEA frequency in both subgroups, nor the association between EpiTrack scores and DDD in the non‐DRE subgroup. As expected, individuals with DRE exhibited lower EpiTrack scores, greater pharmacological burden, and higher epilepsy severity, and consequently, higher IEA frequency. By excluding the individuals with the most severe epilepsy, more marked encephalopathy, and the highest pharmacological burden, the variance of these measures decreases. This reduced variance may explain why the correlations between seizure frequency and epileptiform abnormalities, as well as between EpiTrack scores and DDD, were no longer observed in the non‐DRE group. Conversely, in the DRE subgroup, the absence of correlations between EpiTrack scores and both seizure frequency and IEAs may reflect the small sample size or the finding that, in a condition already characterized by marked epilepsy severity and encephalopathy, factors other than seizures and IEAs strongly associate with cognitive impairment.

Notably, the strong association between depressive symptoms and EpiTrack performance persisted in subgroup analyses (as demonstrated by correlation, ANCOVA, and mediation analyses), confirming its independence from drug resistance. These findings underscore once again the relevance of the link between the severity of depressive symptoms and cognitive outcomes in PwE, regardless of seizure control or epilepsy severity.

### 4.1 Strengths and limitations

From a methodological standpoint, the rigorous and multimodal statistical procedure, the good sample size, and the use of well‐validated scales to measure cognitive performance and psychological variables in PwE are important strengths of our work.

The present study is not free from limitations, mainly linked to the absence of a complete neurocognitive assessment and neuropsychological assessment, the sample bias (a great proportion of PwE in our cohort were affected by DRE), the heterogeneity of sample population, the self‐reported nature of BDI‐II and GAD‐7 scales, and the extreme collinearity of the variables involved in cognitive performance determinations (the latter, an unmodifiable factor of this topic). Moreover, the cross‐sectional nature of the study results in constraints on causal inference.

### 4.2 Future directions

A variety of psychosocial variables, widely interconnected with depressive symptoms, were not included in the present study (such as resilience, and mood‐related and epilepsy constructs). Future studies with ad hoc study design are needed in this field to explore the possible influences, magnitudes, and moderation effects of these variables in promoting epilepsy‐related cognitive burden.

## 5 CONCLUSIONS

More severe depressive symptoms are strongly associated with poorer cognitive performance in epilepsy; this association remains significant after controlling for epilepsy‐related factors (IEAs, seizure frequency, and DDD). The link between depressive symptoms and cognitive impairment could be bidirectional, because depressive symptoms both could predispose to the occurrence of cognitive impairment or could be the direct effect of cognitive dysfunctions in PwE. Screening for depressive symptoms and cognitive dysfunctions should be strongly recommended for global clinicians caring for PwE. Finally, we confirmed the relevant contribution of IEAs, seizures, and pharmacological burden in fostering epilepsy‐related cognitive burden.

## AUTHOR CONTRIBUTIONS

**Biagio Maria Sancetta**: Data curation; formal analysis; investigation; methodology; writing—original draft; writing—review & editing. **Giuli Lippa**: Investigation; data curation; writing—original draft. **Marianna Nesta**: Investigation; data curation; writing—review & editing. **Lorenzo Ricci**: Investigation; supervision; writing—review & editing. **Simona Paola Carbone**: Data curation; writing—review & editing. **Lorenzo Veronese:** Data curation; writing—review & editing. **Giulia Conti**: Writing—review & editing. **Marco Sferruzzi**: Writing—review & editing. **Vincenzo Di Lazzaro**: Visualization; writing—review & editing. **Mario Tombini**: Investigation; resources; supervision; validation; visualization; writing—review & editing. **Giovanni Assenza**: Conceptualization; data curation; investigation; resources; supervision; validation; visualization; writing—review & editing.

## CONFLICT OF INTEREST STATEMENT

None of the authors has any conflict of interest to disclose. We confirm that we have read the Journal's position on issues involved in ethical publication and affirm that this report is consistent with those guidelines.

## Supporting information


FIGURE S1.

FIGURE S2.

FIGURE S3.

FIGURE S4.

DATA S1.

## Data Availability

Data and code may be provided to interested researchers upon reasonable request to the corresponding authors, after clearance from the local ethics committee.
