# Supplementary figures and images for: Depressive symptoms as independent correlates of epilepsy‐related cognitive burden
Source: Epilepsia. 2026 Jan 28;67(5):2425–39. doi: 10.1002/epi.70108 (PMC13179664; doi:10.1002/epi.70108)

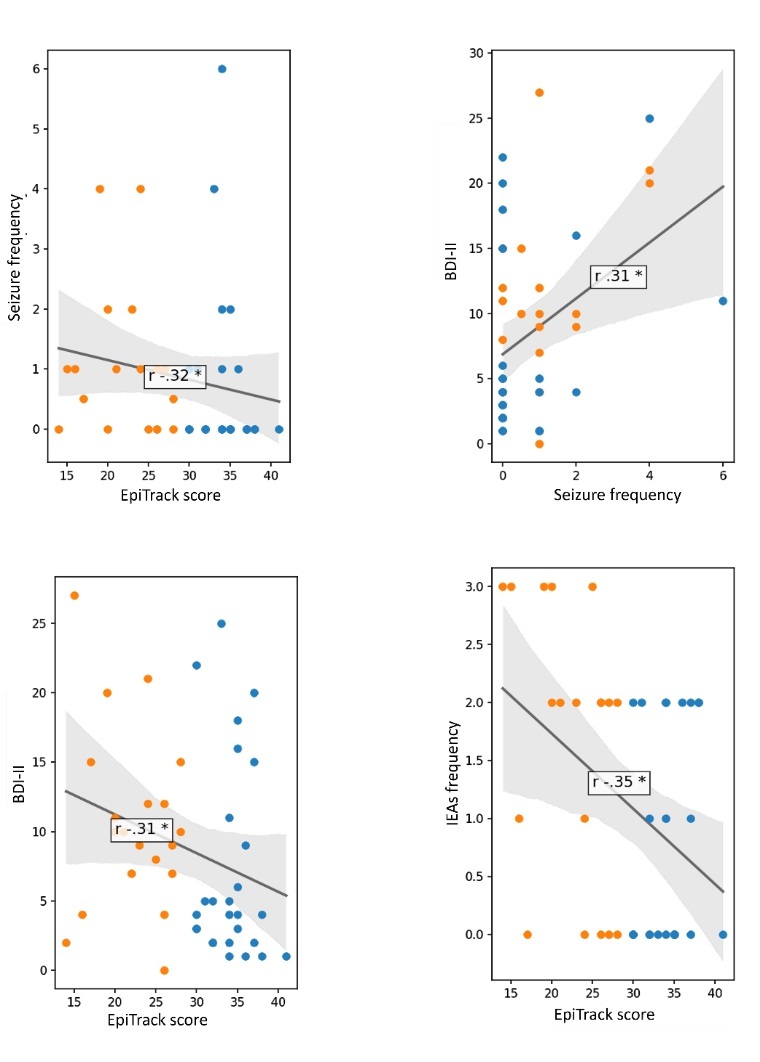

Supplement: Supplementary file 1 — FIGURE S1. [file EPI-67-2425-s003.jpg]

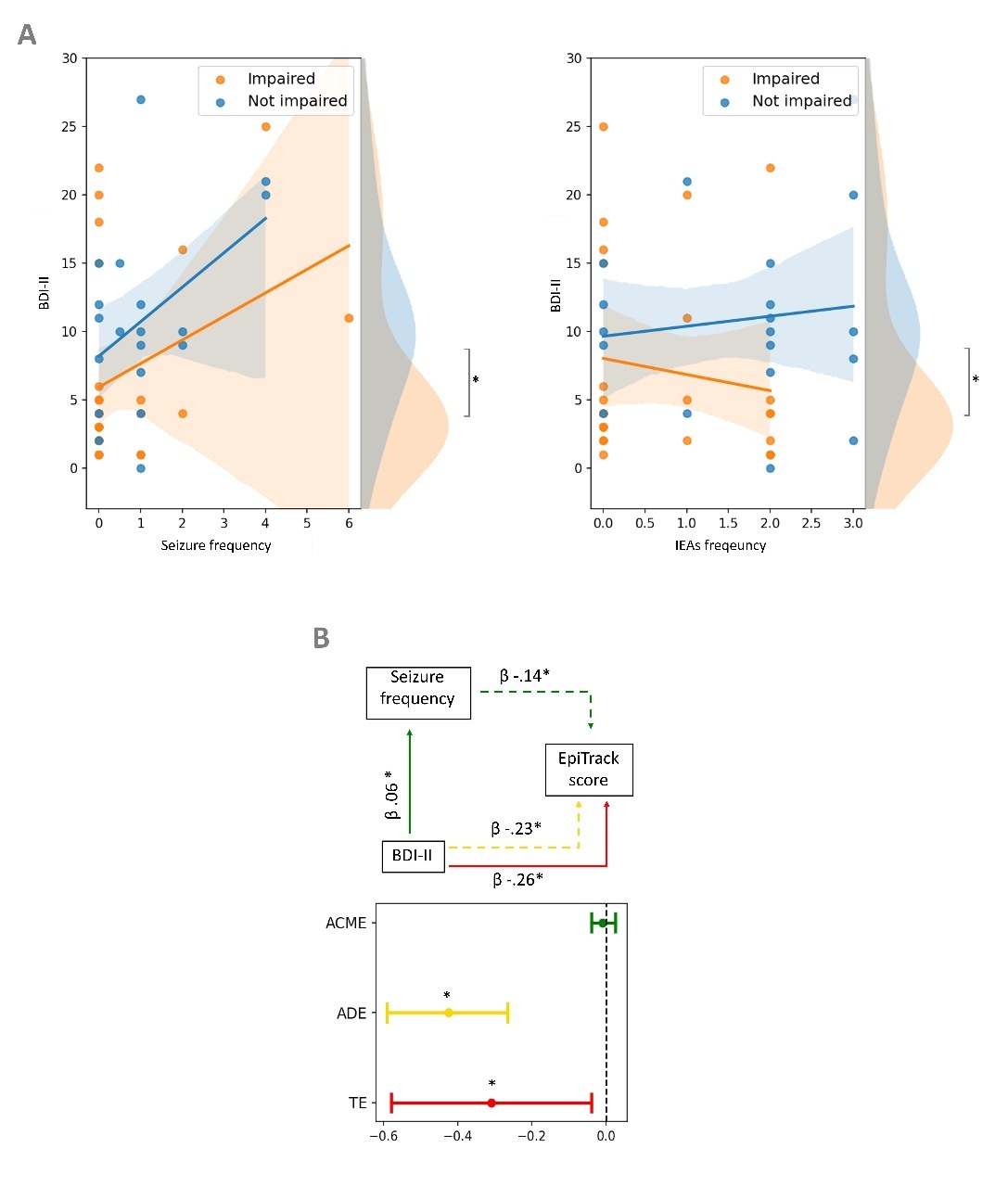

Supplement: Supplementary file 2 — FIGURE S2. [file EPI-67-2425-s001.jpg]

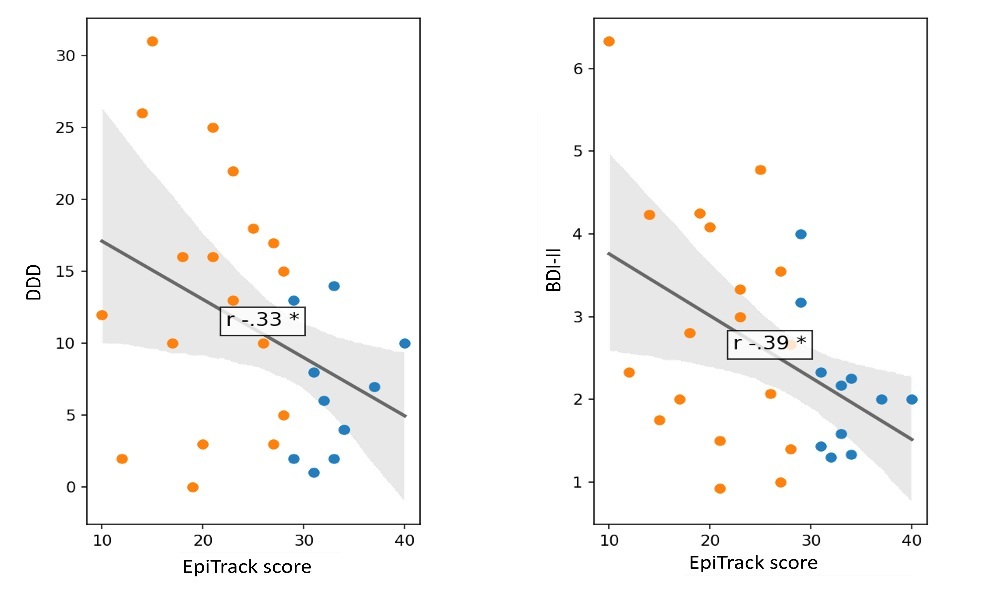

Supplement: Supplementary file 3 — FIGURE S3. [file EPI-67-2425-s002.jpg]

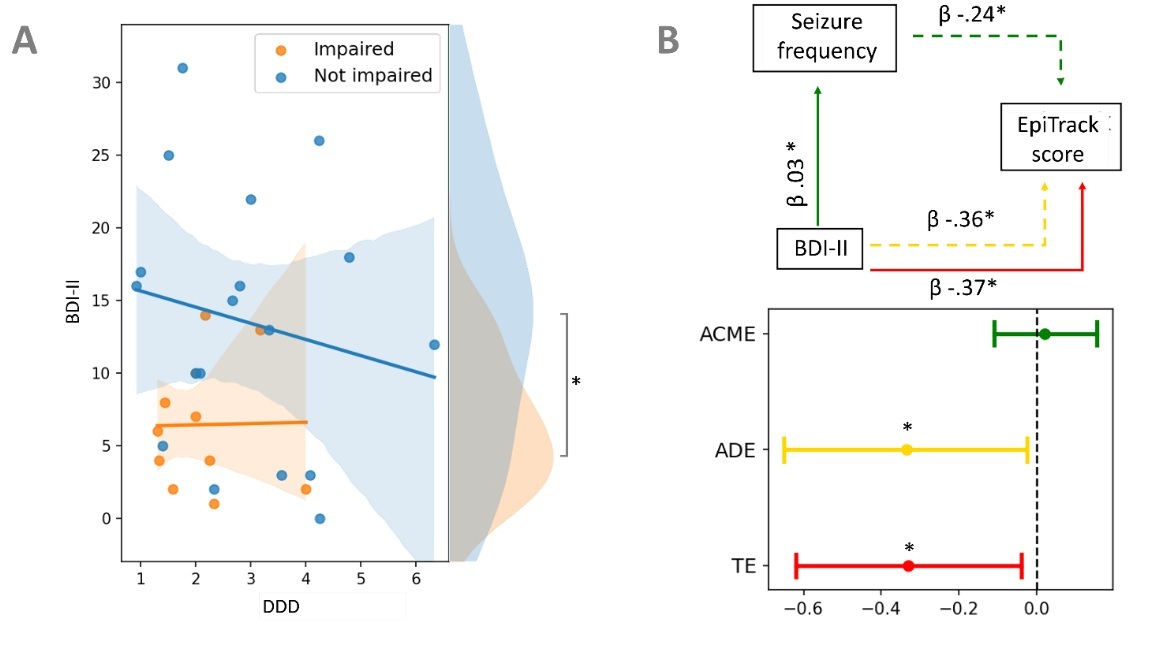

Supplement: Supplementary file 4 — FIGURE S4. [file EPI-67-2425-s004.jpg]
