# Supplementary material for: Depressive symptoms as independent correlates of epilepsy‐related cognitive burden
Source: Epilepsia. 2026 Jan 28;67(5):2425–39. doi: 10.1002/epi.70108 (PMC13179664; doi:10.1002/epi.70108)
Supplement: Supplementary file 5 — DATA S1. [file EPI-67-2425-s005.docx]

**8. Supplementary materials**

- 1. **Subgroup analysis on PwE without DRE**

As shown in *Supplementary Figure 1*, the age-corrected EpiTrack score was inversely correlated with BDI-II (R -.31, p-value .03), seizure frequency (R -.32, p-value .03), IEAs frequency (R -.35, p-value .01, CI []) and ASMs number (R -.27, p-value .04). BDI-II was directly correlated with seizure frequency (R .31, p-value .03). BDI-II was not statistically correlated with DDD or IEAs frequency. GAD-7 was not statistically correlated with the EpiTrack score.

As shown in *Supplementary Figure 2A*, ANCOVA showed a significant between-subject factor influence on BDI-II variance both when covariates (IEAs and seizure frequency) were added altogether in the model (F-statistic 4.49, p-value 0.04) and separately (IEAs frequency: F-statistic 5.31, p-value .03; seizure frequency: F-statistic 6.49, p-value .01).

Concerning moderation analysis, as shown in *Supplementary Figure 2B*, BDI-II showed a significant TE on EpiTrack (p-value .03); BDI-II effect on EpiTrack score remained significant when accounting for seizure frequency effect (ADE p-value.04, respectively); BDI-II effect on EpiTrack score was not moderated by an indirect influence of seizure frequency.

- 1. **Subgroup analysis on PwE with DRE**

As shown in *Supplementary Figure 3*, the age-corrected EpiTrack score was inversely correlated with DDD (R -.39, p-value .03), BDI-II (R -.33, p-value .04) and ASMs number (R -.34, p-value .04). BDI-II was not statistically correlated with DDD, seizure frequency and IEAs frequency. GAD-7 was not statistically correlated with the EpiTrack score.

As shown in *Supplementary Figure 4A*, ANCOVA (DDD as covariate) showed a significant between-subject factor influence on BDI-II variance (F-statistic 6.55, p-value 0.01).

Concerning moderation analysis, as shown in *Supplementary Figure 2B*, BDI-II showed a significant TE on EpiTrack (p-value .02); BDI-II effect on EpiTrack score remained significant when accounting for seizure frequency effect (ADE p-value .01, respectively); BDI-II effect on EpiTrack score was not moderated by an indirect influence of seizure frequency.
